# Bayesian frailty modeling of correlated survival data with application to under-five mortality

**DOI:** 10.1186/s12889-020-09328-7

**Published:** 2020-09-21

**Authors:** Refah M. Alotaibi, Hoda Ragab Rezk, Chris Guure

**Affiliations:** 1 Mathematical Sciences Department, College of Science, Princess Nourah bint Abdulrahman University, P.O. Box 84428, Riyadh, 11671 Saudi Arabia; 2 Department of statistics, Al-Azhar University, Cairo, Egypt; 3 Department of Biostatistics, School of Public Health, University of Ghana, Accra, Ghana

**Keywords:** Bayesian approach, Frailty models, Correlated data, Community frailty, Under-five mortality, Parametric regression models, Deviance information criteria, Bayes factor

## Abstract

**Background:**

There is high rate of under-five mortality in West Africa with little effort made to study determinants that significantly increase or decrease its risk across the West African sub-region. This is important since it will help in the design of effective intervention programs for each country or the entire region. The overall objective of this research evaluates the determinants of under-five mortality prior to the end of the 2015 Millennium Development Goals, to guide West African countries implement strategies that will aid them achieve the Sustainable Development Goal 3 by 2030.

**Method:**

This study used the Demographic and Health Survey (DHS) data from twelve (12) out of the eighteen West African countries; Ghana, Benin, Cote d’ Ivoire, Guinea, Liberia, Mali, Niger, Nigeria, Sierra Leone, Burkina Faso, Gambia and Togo. Data were extracted from the children and women of reproductive age files as provided in the DHS report. The response or outcome variable of interest is under-five mortality rate. A Bayesian exponential, Weibull and Gompertz regression models via a gamma shared frailty model were used for the analysis. The deviance information criteria and Bayes factors were used to discriminate between models. These analyses were carried out using Stata version 15 software.

**Results:**

The study recorded 101 (95% CI: 98.6–103.5) deaths per 1000 live births occurring among the twelve countries. Burkina Faso (124.4), Cote D’lvoire (110.1), Guinea (116.4), Nigeria (120.6) and Niger (118.3) recorded the highest child under-5 mortality rate. Gambia (48.1), Ghana (60.1) and Benin (70.4) recorded the least unde-5 mortality rate per 1000 livebirths. Multiple birth children were about two times more likely to die compared to singleton birth, in all except Gambia, Nigeria and Sierra Leone. We observed significantly higher hazard rates for male compared to female children in the combined data analysis (HR: 1.14, 95% CI: [1.10–1.18]). The country specific analysis in Benin, Cote D’lvoire, Guinea, Liberia, Mali and Nigeria showed higher under-5 mortality hazard rates among male children compared to female children whilst Niger was the only country to report significantly lower hazard rate of males compared to females.

**Conclusion:**

There is still quite a substantial amount of work to be done in order to meet the Sustainable Development Goal 3 in 2030 in West Africa. There exist variant differences among some of the countries with respect to mortality rates and determinants which require different interventions and policy decisions.

## Background

Under-five mortality is defined as the probability of a child dying before the fifth birthday or before reaching the age of five [1]. Approximately 9 million deaths occur per year worldwide that are attributable to under-five mortality [2]. This has brought about major concerns and efforts to reduce under-five mortality, as child health is a key indicator of economic development [3]. Thus, in 2000, world leaders from 189 countries came together and developed eight Millennium Development Goals (MDG) which were to be achieved by end of year 2015. The MDG 4 called for a reduction in under-five mortality by two thirds [4, 5]. Out of the eight MDGs, four of them were directly related to child mortality [3].

The twentieth century experienced a severe decline in under-five mortality in most countries, irrespective of their socio-economic and development status [6]. Yet the gap between developed and developing nations in child mortality is still high as children in developing countries are 10 times more likely to die before the age of five [2]. Studies reveal that the global under-five mortality has reduced from 91 deaths per 1000 live births in 1990 to about 43 deaths per 1000 live births in 2015 [7]. Despite this substantial progress, it is projected that about 68.8 million children will die before their fifth birthday between 2016 and 2030 if the mortality rate in 2015 remains constant [4, 5]. Thus, there is still a need for all countries to continue to work towards reducing under-five mortality.

Under-five mortality remains a great concern in many countries. Sub-Saharan Africa has the highest under-five mortality rate in the world. Through efforts and collaborations, Ghana experienced a 58% reduction in under-five mortality from 127 deaths per 1000 live births in 1990 to 62 deaths per 1000 live births in 2015. Others include Mali (115), Guinea (94), Sierra Leone (120), Liberia (70), Cote d’ Ivoire (93), Togo (78), Benin (100), Nigeria (109), Gambia (69) and Burkina Faso (89 per 1000 deaths live births of under-fives [4, 5]. Though this is a substantial progress, all these West African states failed to meet the 2015 MDG of 40 deaths per 1000 livebirths [8].

There are regional and socioeconomic inequalities in under-five mortality within countries. For under-five mortality to be reduced effectively, determinants of high mortality among disadvantaged people, communities and regions need to first of all be identified [9].

It is anticipated that, the high prevalence of under-five mortality could be due to unobserved differences that exist between communities. There are substantial regional disparities in under-five mortality, and evidence suggests that both individual and community level characteristics have an influence on health outcomes [7]. Studies have been conducted on under-five mortality worldwide, see for instance Kayode et al. 2012 [2], You et al 2015 [4, 5], Deribew et al. 2007 [6] and Rudan et al. 2008 [10].

It has been established that cluster levels (community effects) have significant effects on under-five mortality [7]. Unfortunately, community effects are usually not considered nor accounted for in studies that seek to find the determinants of under-five mortality [11]. However, child survival and under-five mortality have only recently been analyzed using frailty models [11]. The ability to assess and account for cluster level variations of under-five mortality using a time-to-event model, will help to re-evaluate current policies that target reduction of under -five mortality in the West African sub-region.

Cox proportional hazards model proposed by Cox, [12] is by far the most popular regression model in analyzing time-to-event data. This model has been implemented via the frequentist or the Bayesian frameworks, refer to, Austin, 2017 [13], Clayton et al., 1985 [14], Clayton 1991 [15], Duchateau et al., 2007 [16], Koissi et al., 2013 [17], Tsonaka et al. 2009 [18] and Van Oirbeek et al., 2010 [19] for further information. Though, highly used by both researchers and practitioners, the model requires that the survival times for subjects be independent and identically distributed [16, 19, 20]. An assumption that may not be practically attainable in all situations, since some subjects may be related either by virtue of their relation within a family [21], or by shared community or environment [22]. Factors of this nature represent the cumulative effect of unobserved or unmeasured covariates that may reflect impacts of environmental and socio-cultural factors [21]. There are a number of biostatistical methods used to quantify the size of the effect of unobserved factors which may act either multiplicatively or additively on the baseline hazards Duchateau et al., [16]; Spizzichino [23]. Breslow & Clayton [24], Hougaard [25], and Klein [26], modelled the dependence of the covariate structure via frailty terms of an assumed parametric distribution.

Most often than not, researchers unjustifiably assume that a particular distribution is appropriate for their data without any scientific or empirical evidence but on the bases of tractability of the frailty function and availability of software [27]. This does not permit appropriate inference to correctly inform appropriate interventions. The objectives of this study are three fold, 1) to test how well a proposed distribution fits the data at hand before any inference can be drawn, 2) to account or quantify the amount of heterogeneity that exists at community level that may bias posterior mean estimates and their corresponding posterior standard deviations and consequently the credible intervals, and 3) to determine consistent determinants of under-five mortality across all the twelve countries in the West African sub-region. The overall objective of this research is to evaluate the determinants of under-five mortality prior to the end of the 2015 Millennium Development Goals, to guide West African countries implement strategies that will aid in the achievement of the Sustainable Development Goal 3 by 2030.

## Methods

### Source of data

This study obtained and analyzed data from the Demographic and Health Surveys (DHS) across all the participating twelve countries in West Africa these are; Ghana, Benin, Cote d’ Ivoire, Guinea, Liberia, Mali, Niger, Nigeria, Sierra Leone, Burkina Faso, Gambia and Togo. Countries that were included in this study had their DHS conducted between the years 2012 to 2014. Countries that collected their data before 2012 and after 2014 were excluded in this analysis. This study was considered only in West Africa due to its relatively high rates of under-five mortality in the African Continent and the World as a whole. The DHS conducted across some selected countries in the world are based on nationally representative samples from all the participating countries. The collection of this data across West African countries use a similar selection approach via stratified two-stage sampling techniques. The information gathering approach is via structured interviews administered by well-trained research assistants or field workers. Data used in this study were extracted from the child and women’s questionnaire which contains information from 5 years prior to the survey date and questions about maternal factors, birth history and community factors as well. Country specific samples were; Burkina Faso (15,162), Benin (12,290), Cote D’Ivoire (7149), Ghana (5675), Gambia (7798), Guinea (6977), Liberia (6432), Mali (9964), Nigeria (30,540), Niger (12,767), Sierra Leone (8359) and Togo (6581). The overall combined sample is 129,693.

### Study outcome

The main outcome for this study was time-to-under-five mortality (death occurring to a child before he/she reaches the age of five) among twelve different countries in the West African sub-Region. Under-five mortality is defined as mortality occurring from the age of zero months to 59 months. Therefore, the dependent variable in this study was defined as “the risk of death occurring between 0 to 59 months period. The outcome variable was thus survival time in months for the children under the age of five. Children who died under the age of five were deemed to have had the event and assigned the number 1. Those who did not die within the period were censored and assigned the number 0. This study allowed us to determine whether factors that have either positive or negative effects in under-fives are similar or different across the region. Women were asked about the age of children, including (month and year) birth of a child born alive, sex of the child and whether the child was still alive or dead. With dead children, information from their mothers regarding age at death was also obtained. Stillbirth or miscarriages were not included in this study.

### Study setting

#### Explanatory variables

The exposure variables that were of interest and considered in the analysis are; mother’s age group (15–19, 20–24, 25–29, 30–34, 35–39, 40–44, 45–49), type of residence (urban, rural), mother’s level of education (No education, Primary, Secondary and Higher), birth status (singleton birth, multiple births), sex of the child (male, female), wealth index (poorest, poorer, middle, rich, richer), birth order (1st - 3rd, 4th - 6th, 7th + children), religion (Christian, Muslim, No religion, other religion and Traditional), place of delivery (home vs health facility), mode of delivery (caesarean section or no caesarean section), weight of the child (small (less than 2.5 kg), average (2.5 kg < =weight < =4.5 kg) and large (> 4.5 kg)) these were birth weights recorded for children 5 years preceding the survey from written records or mother’s recall of the size of the child at birth and preceding birth interval (< 12 months, 11 to 23 months, 24 to 35 months, > 35 months).

Some of the included independent variables in the model were selected based on their significance at the bivariate level (hazard ratios of variables that did not have 1 included in their credible intervals) whilst others were based on recommendation from literature. We checked for a 2-way interaction effect using all possible combinations of the exposure variables via a two-model approach, that is a model with the interaction effect referred to as “full model” and another without the interaction effect called “half model”. After which, we run a likelihood ratio test (in that the half_model was nested within the full_model) and using the *p*-values with reference to an alpha level of 0.05, the model with interactions was rejected because it was not significant.

#### Analytical procedure

A Bayesian parametric proportional hazards modeling approach was adopted for this study. We looked at the effects of specifying different models with or without a frailty term on the distribution of under-five mortality rate estimates for each country and the combined data from all the countries. Frailties were modelled according to the number of regions (following DHS classification) of the country and further into whether the respondents were either residing in a rural or urban setting. For instance, Ghana had 10 regions at the time of the survey and so within a region, respondents were either residing in a rural community or urban. Therefore, Ghana had 20 strata. The rest are as follows; Burkina Faso 26, Benin 23, Cote D’ Ivoire 21, Gambia 14, Guinea 15, Liberia 10, Mali 11, Nigeria 12, Niger 15, Sierra Leone 8 and Togo 11 strata. The frailty was specified to control for the heterogeneity between residence and across regions [13]. We specified three different distributional (the exponential, Weibull and Gompertz) forms for the hazard function in two different dimensions. One dimension assumes that community level variations are constant or do not vary and therefore, there is no heterogeneity between groups. The second dimension assumes no heterogeneity within (clusters or community) groups but between (clusters or community) groups and so a shared (gamma) frailty model is specified. Therefore, in the first set of the models, it is assumed that community level effects are not of particular interest and therefore the data follows either the standard exponential, Weibull or Gompertz regression model. In the second stage, we assumed a variation between communities and therefore made use of a frailty term to account for the variations using the parametric proportional hazards model framework as specified above.

The Cox proportional hazards regression is one of the popular statistical models used in analyzing censored survival data. The Cox model does not assume any specific form of the baseline hazard function, as an alternative to the Cox model, one can make assumptions about the shape of the underlying hazard function by using a parametric model; parametric models directly estimate absolute effects in addition to relative effects [28]. The hazard function is often of fundamental interest since it represents an important aspect of the time course of the disease in question [29]. Due to our interest in estimating whether the hazards of death in under-fives among the twelve countries is either decreasing, increasing or constant, we made use of only parametric proportional regression models. One of the advantages of the parametric models is that, there are better fit models over Cox when the shape of the hazard is known.

There were six models specified for this work. The first three were Bayesian regression models (exponential, Weibull and Gompertz) specified and fitted with the assumption that community heterogeneity (frailty) was insignificant. The second three Bayesian regression models, same as above, include a gamma shared frailty term with the assumption of a significant unobserved effect (presence of heterogeneity). Analysis were carried out on each model via the Bayesian approach for all the data sets. Comparison of the models were carried out using the deviance information criteria (DIC) and the Bayes factors (BF). The DIC is the Bayesian version of the frequentist AIC and BIC. It has two components, the goodness of fit represented by $\overline{D}\left(\theta \right)$ and the model complexity term *pD*. This in effect makes $DIC=\overline{D}\left(\theta \right)+ pD$. Smaller values of the DIC are more preferable to larger values. The Bayes Factor relies on the expression that, the posterior odds are a product of the prior odds and the BF. If we assume that two models are equally probable, then the posterior odds will be equal to that of the Bayes Factor. Therefore, a model with a Bayes Factor > 1 compared to the other is more preferable. These analyses were carried out using Stata version 15 software.

#### Test of proportionality under survival analysis

Schoenfeld residual test and a graphical approach were used to test for the proportional hazard’s assumption conditions. The Schoenfeld test hypothesizes that some variables do not vary with time. This hypothesis implies that variables remain constant over the study period and therefore satisfy the proportionality assumption under the PH model.

### Models with and without frailty terms

#### The proportional hazards model without a frailty term

The proportional hazards model specifies that the hazard at some time *t* for an individual with covariate *x* can be expressed as

1 $$ h\left(t|x\right)={h}_0(t)\exp \left(\overset{`}{X}\beta \right) $$

where *h*_0_(*t*) is the baseline hazard function, *X*^’^ represents the vector of covariates, *β* the regression coefficients and *S*_0_(*t*_*i*_) the survival function.

The likelihood function *L*(*D*| *h*_0_(*t*), *β*) that can be expressed in the form of a right censored data (for the under-five mortality) on *n* number of subjects is

2 $$ L\left(D|{h}_0(t),\beta \right)={\prod}_{i=1}^n{\left\{{h}_0\left({t}_i\right)\mathit{\exp}\left({\overset{`}{X}}_i\beta \right)\right\}}^{\delta_i}\left({S}_0{\left({t}_i\right)}^{\mathit{\exp}\left({\overset{`}{X}}_i\beta \right)}\right) $$

#### The proportional hazards model with a frailty term

In this analysis, we specify a shared frailty model which implies that similar observations within a group have similar characteristics or frailty but these frailties differ between groups. Frailty models in survival analysis account for unobserved heterogeneity that occurs because some observations are more failure-prone and therefore, more “frail” than other observations. We assume that the survival times for say the *i*^*th*^ subject (*i* = 1 . . . *n*) in the *j*^*th*^ group (*j* = 1 . . . *m*) is denoted by *T*_*ij*_ with an unobserved frailty parameter given as *u*_*i*_ (for the *j*^*th*^ group). With this, the hazard function for the proportional hazards model is given as

3 $$ h\left({t}_{ij}|{X}_{ij},{u}_j\right)={h}_0\left({t}_{ij}\right)\exp \left({\overset{`}{X}}_{ij}\beta \right){u}_j $$

where *u*_1_, . . ., *u*_*m*_ represent the frailty and *h*_0_(*t*), *X*_*ij*_ and *β* are the baseline hazards, vector of covariates and regression coefficients respectively. The *u*_*j*_’s are independently and identically distributed with mean 1 and variance *θ*. The frailty distribution for each of *u*_*j*_ is assumed to be independent gamma following Clayton [12] and given as

4 $$ {u}_j\sim Gamma\left(\eta, \eta \right),j=1,\dots, m $$

where *η* is the unknown variance of *u*_*j*_. We specify the following distribution for the frailty, which is

$$ X\sim Gamma\left(a,b\right)\propto {x}^{a-1}\exp \left(- bx\right),\mathrm{for}\ x>0,a>0\ \mathrm{and}\ b>0 $$

### Description of exponential, Weibull and Gompertz distributions

#### Exponential and Weibull distributions

The exponential distribution is a special case of the Weibull distribution, that is suitable for modeling data with constant hazard. In other words, the hazards of the exponential distribution of an event occurring is constant. The Weibull distribution is more suitable for modeling data with monotone hazard rates that are either increasing or decreasing exponentially with time.

The hazard and survival functions of the Weibull distribution are

$$ h(t)= p\alpha {t}^{p-1} $$

5 $$ S(t)=\exp \left(-\alpha {t}^p\right) $$

If *p* = 1, the hazard and survival function of the Weibull distribution as described in eq. (5) reduces to that of the exponential. The parameter *α* is known as the scale parameter of the Weibull distribution. This parameter is parametrized for both exponential and Weibull regression models as

6 $$ {\alpha}_j=\exp \left({X}_j\beta \right) $$

This expression, eq. (6) is similar to that given in eq. (1). In this case, there is no auxiliary variable for the exponential distribution but for the Weibull which is the shape parameter (*p*).

Therefore, the proportional hazards models as described in eq. (3) if specified for the exponential and Weibull distributions have their baseline hazards given respectively as

$$ {h}_0(t)=1 $$

7 $$ {h}_0(t)=p{t}^{p-1} $$

where *p* is the shape parameter estimated from the data.

#### Gompertz distribution

The Gompertz distribution has been extensively used in the medical field for modeling mortality data. Like the Weibull distribution, the Gompertz is also a two-parameter distribution. The hazard and survival functions of the Gompertz distribution are

$$ h(t)=\alpha\ \exp \left(\gamma t\right) $$

8 $$ S(t)=\mathit{\exp}\left\{-\alpha \gamma \left(\gamma t-1\right)\right\} $$

The baseline hazards for the Gompertz regression model is

9 $$ {h}_0(t)=\exp \left(\upgamma t\right) $$

where *γ* is an auxiliary parameter estimated from the data.

When the auxiliary parameter (*γ*) is positive, its hazard function increases with time but if negative, it decreases with time. It is worth mentioning that if *γ* is zero, the hazard function is reduced to the exponential.

#### Bayesian proportional hazards model with/without a frailty term

The posterior probability density function which summarizes our beliefs about a particular parameter is obtained via the Bayes’ rule as

10 $$ \pi\ \left(\theta |D\right)=\frac{\pi \left(\theta \right)L\left(D|\theta \right)}{\int_{\Theta}\pi \left(\theta \right)L\left(D|\theta \right) d\theta} $$

Which can be summarized as

11 $$ \pi \left(\theta |D\right)\propto \pi \left(\theta \right)L\left(D|\theta \right) $$

Therefore, the posterior distribution can be obtained from eq. (11) as

12 $$ \pi \left({h}_0(t),\beta |D\right)\propto {\prod}_{i=1}^n{\left\{{h}_0\left({t}_i\right)\mathit{\exp}\left({\overset{`}{X}}_i\beta \right){u}_i\right\}}^{\delta_i}\left({S}_0{\left({t}_i\right)}^{\mathit{\exp}\left({\overset{`}{X}}_i\beta \right)}\right)\pi \left(\beta \right) $$

where the baseline hazards function *h*_0_(*t*_*i*_) as provided in eq. (12) takes the form 1, *pt*^*p* − 1^ and exp(*γt*) for the exponential, Weibull and Gompertz distribution respectively. We specified normal distribution with mean *μ*_0_ = 0 and variance ${\sigma}_0^2=100$ as priors for the regression coefficients *βs* with a probability density function

13 $$ f\left(x|\ {\mu}_0,{\sigma}_0^2\right)=\frac{1}{\sqrt{2\pi {\sigma}_0^2}}{e}^{-\frac{{\left(x-{\mu}_0\right)}^2\ }{2{\sigma_0}^2}} $$

In analyzing the frailty parameter (*u*) via the Bayesian approach, we adopt a gamma distribution with mean = 1 and a variance = 1000 which is a conjugate prior for the hyperparameters *η*.

## Results

### Descriptive characteristics of the study participants

The distribution of background characteristics of study respondents are presented in Table 1. A total of 129,693 children under-5 years were represented in the study. The mean age of the mothers interviewed was approximately 29.1 years and the mean age at first birth was approximately 19.1 years. Mothers within the age group of 20–24, 25–29 and 30–34 years recorded the highest number of respondents. Similar pattern of the mother’s age was observed for each of the twelve West African countries. This indicate that younger women were the most represented in the study.

**Table 1:** Background characteristics of study respondents (*n* = 129,693) presented in means and percentages for overall data and country specific for all variables

**Characteristics**	**Burkina Faso**	**Benin**	**Cote D’Ivoire**	**Ghana**	**Gambia**	**Guinea**	**Liberia**
**Total**	**15,162**	**12,290**	**7149**	**5675**	**7798**	**6977**	**6432**
**Mothers’ characteristic**							
**Current age (mean + S.E)**	29.2 ± 0.08	29.27 ± 0.08	28.65 ± 0.13	30.54 ± 0.14	29.17 ± 0.13	28.48 ± 0.14	28.37 ± 0.16
** Current age group**							
15–19	707 (4.66)	415 (3.37)	534 (7.47)	203 (3.57)	380 (4.87)	670 (9.61)	599 (9.32)
20–24	3617 (23.85)	2293 (18.66)	1669 (23.35)	968 (17.06)	1657 (21.25)	1582 (22.68)	1645 (25.57)
25–29	3978 (26.23)	3839 (31.24)	1994 (27.89)	1419 (25.01)	2218 (28.44)	1797 (25.75)	1667 (25.92)
30–34	3304 (21.79)	3021 (24.58)	1459 (20.42)	1413 (24.9)	1787 (22.91)	1254 (17.98)	1115 (17.33)
35–39	2146 (14.16)	1743 (14.18)	905 (12.66)	1050 (18.51)	1086 (13.93)	1014 (14.54)	877 (13.63)
40–44	1102 (7.27)	752 (6.12)	452 (6.32)	482 (8.5)	534 (6.84)	495 (7.09)	393 (6.11)
45–49	309 (2.04)	228 (1.85)	135 (1.89)	139 (2.45)	136 (1.75)	164 (2.35)	136 (2.12)
** Age at first birth (mean + S.E)**	18.89 ± 0.04	20.01 ± 0.06	18.77 ± 0.08	20.75 ± 0.12	19.31 ± 0.08	18.15 ± 0.07	18.49 ± 0.07
** Age at first birth group**							
< 15	442 (2.92)	844 (6.87)	560 (7.83)	175 (3.08)	625 (8.01)	804 (11.53)	439 (6.82)
15–19	9448 (62.32)	5274 (42.92)	4037 (56.46)	2371 (41.78)	3847 (49.34)	4168 (59.74)	4010 (62.34)
20–24	4507 (29.73)	4446 (36.18)	2069 (28.94)	2089 (36.81)	2557 (32.79)	1572 (22.53)	1618 (25.16)
25–29	653 (4.31)	1386 (11.28)	418 (5.84)	798 (14.06)	651 (8.35)	340 (4.87)	318 (4.94)
30–34	96 (0.63)	272 (2.21)	50 (0.7)	208 (3.67)	105 (1.35)	78 (1.12)	43 (0.67)
> 34	15 (0.1)	67 (0.54)	16 (0.23)	34 (0.6)	12 (0.15)	15 (0.21)	4 (0.07)
** Highest educational level**							
No education	12,784 (84.32)	8717 (70.93)	4544 (63.56)	1552 (27.36)	4648 (59.61)	5437 (77.92)	2680 (41.66)
Primary	1610 (10.62)	2068 (16.83)	1869 (26.15)	1141 (20.1)	1122 (14.39)	843 (12.09)	1960 (30.47)
Secondary	695 (4.59)	1372 (11.16)	662 (9.26)	2730 (48.1)	1777 (22.79)	609 (8.73)	1610 (25.02)
Higher	72 (0.48)	133 (1.08)	74 (1.03)	252 (4.44)	251 (3.21)	88 (1.26)	183 (2.85)
** Current employment status**							
Not currently employed	3427 (22.6)	3760 (30.6)	2085 (29.16)	1174 (20.68)	4060 (52.07)	1418 (20.33)	2745 (42.68)
Currently employed	11,735 (77.4)	8529 (69.4)	5064 (70.84)	4501 (79.32)	3738 (47.93)	5559 (79.67)	3687 (57.32)
** Marital status**							
Not current married	410 (2.7)	702 (5.71)	1081 (15.13)	814 (14.35)	496 (6.37)	495 (7.09)	1572 (24.44)
Currently married/union	14,752 (97.3)	11,588 (94.29)	6068 (84.87)	4861 (85.65)	7301 (93.63)	6482 (92.91)	4860 (75.56)
** Residence**							
Urban	2546 (16.79)	4995 (40.64)	2688 (37.6)	2553 (44.99)	3724 (47.76)	1832 (26.26)	3207 (49.85)
Rural	12,616 (83.21)	7295 (59.36)	4461 (62.4)	3122 (55.01)	4074 (52.24)	5144 (73.74)	3226 (50.15)
** Wealth**							
Poorest	3142 (20.72)	2536 (20.64)	1770 (24.76)	1258 (22.17)	1566 (20.09)	1569 (22.49)	1557 (24.2)
Poorer	3317 (21.87)	2451 (19.94)	1592 (22.27)	1193 (21.03)	1715 (21.99)	1535 (21.99)	1438 (22.36)
Middle	3283 (21.66)	2417 (19.67)	1360 (19.03)	1111 (19.57)	1560 (20)	1433 (20.53)	1358 (21.12)
Richer	3147 (20.76)	2469 (20.09)	1346 (18.82)	1069 (18.84)	1584 (20.32)	1371 (19.65)	1217 (18.92)
Richest	2273 (14.99)	2416 (19.66)	1081 (15.13)	1044 (18.4)	1373 (17.6)	1070 (15.33)	862 (13.4)
** Religion**							
Christian	4033 (26.6)	6907 (56.2)	2876 (40.23)	4291 (75.61)	188 (2.41)	545 (7.81)	5428 (84.38)
Muslim	9678 (63.83)	2916 (23.73)	2986 (41.76)	964 (16.99)	7609 (97.58)	6126 (87.81)	749 (11.65)
No religion	158 (1.04)	626 (5.09)	1002 (14.01)	236 (4.16)	0 (0.01)	283 (4.05)	219 (3.41)
Other religion	1 (0.01)	1554 (12.65)	286 (4)	–	–	23 (0.33)	1 (0.01)
Traditional	1291 (8.51)	287 (2.33)	–	183 (3.23)	–	–	35 (0.55)
**Child characteristics**							
**Birth type**							
Singleton	14,576 (96.14)	11,686 (95.09)	6815 (95.33)	5388 (94.94)	7538 (96.67)	6673 (95.65)	6197 (96.33)
Multiple	586 (3.86)	604 (4.91)	334 (4.67)	287 (5.06)	260 (3.33)	304 (4.35)	236 (3.67)
**Sex of child**							
Male	7683 (50.67)	6322 (51.45)	3601 (50.37)	2962 (52.2)	3965 (50.85)	3576 (51.25)	3284 (51.05)
Female	7479 (49.33)	5967 (48.55)	3548 (49.63)	2713 (47.8)	3833 (49.15)	3401 (48.75)	3149 (48.95)
**Child size at birth**							
Large	4629 (30.53)	2215 (18.02)	3552 (49.68)	2899 (51.09)	3964 (50.84)	3372 (48.33)	2973 (46.23)
Average	8534 (56.28)	8408 (68.41)	2475 (34.62)	1880 (33.13)	2173 (27.86)	2706 (38.79)	2182 (33.92)
Small	1999 (13.18)	1667 (13.57)	1122 (15.69)	896 (15.78)	1661 (21.3)	899 (12.89)	1277 (19.85)
**Birth order**							
1st-3rd	7813 (51.53)	7345 (59.76)	4215 (58.96)	3539 (62.35)	4334 (55.58)	3818 (54.72)	3857 (59.96)
4th–6th	5058 (33.36)	3952 (32.16)	2103 (29.41)	1692 (29.82)	2483 (31.84)	2242 (32.13)	1833 (28.5)
7th + chi	2291 (15.11)	993 (8.08)	831 (11.63)	444 (7.83)	981 (12.58)	917 (13.15)	743 (11.54)
**Preceding birth interval**							
< 12 months	2848 (18.78)	2695 (21.93)	1678 (23.47)	1393 (24.55)	1730 (22.19)	1586 (22.73)	1633 (25.39)
11 to 23 months	1531 (10.1)	1412 (11.49)	755 (10.57)	550 (9.7)	856 (10.98)	663 (9.5)	705 (10.96)
24 to 35 months	4660 (30.74)	3416 (27.8)	1806 (25.27)	1297 (22.86)	2520 (32.32)	1885 (27.02)	1496 (23.25)
> 35 months	6123 (40.38)	4766 (38.78)	2909 (40.69)	2434 (42.89)	2691 (34.51)	2843 (40.75)	2599 (40.4)
**Delivery type**							
Skilled delivery	10,099 (66.61)	10,888 (88.6)	4206 (58.84)	4147 (73.07)	4905 (62.91)	2836 (40.65)	3580 (55.65)
Unskilled delivery	5062 (33.39)	1401 (11.4)	2943 (41.16)	1528 (26.93)	2893 (37.09)	4141 (59.35)	2853 (44.35)
**Delivery mode**							
SVD	14,871 (98.08)	11,597 (94.37)	6951 (97.23)	4954 (87.3)	7641 (97.99)	6806 (97.56)	6185 (96.16)
Caesarea	291 (1.92)	692 (5.63)	198 (2.77)	721 (12.7)	157 (2.01)	170 (2.44)	247 (3.84)
**Death status**							
Alive	13,828 (91.2)	11,725 (95.4)	6567 (91.86)	5423 (95.56)	7498 (96.15)	6386 (91.53)	5988 (93.09)
Dead	1334 (8.8)	565 (4.6)	582 (8.14)	252 (4.44)	300 (3.85)	591 (8.47)	444 (6.91)
**Characteristics**	**Mali**	**Nigeria**	**Niger**	**Sierra Leone**	**Togo**	**Overall**	
**Total**	**9964**	**30,540**	**12,767**	**8359**	**6581**	**129,693**	
**Mothers’ characteristic**							
**Current age (mean + S.E)**	28.51 ± 0.1	29.38 ± 0.07	28.88 ± 0.11	28.91 ± 0.12	30.07 ± 0.11	29.13 ± 0.03	
** Current age group**							
15–19	739 (7.42)	1531 (5.01)	728 (5.7)	830 (9.93)	240 (3.64)	7577 (5.84)	
20–24	2112 (21.2)	5961 (19.52)	2714 (21.26)	1696 (20.29)	1150 (17.48)	27,064 (20.87)	
25–29	2846 (28.57)	8510 (27.86)	3534 (27.68)	2085 (24.94)	1857 (28.22)	35,744 (27.56)	
30–34	2128 (21.36)	6702 (21.95)	2805 (21.97)	1586 (18.97)	1540 (23.41)	28,114 (21.68)	
35–39	1347 (13.52)	4747 (15.54)	1822 (14.27)	1313 (15.71)	1101 (16.74)	19,152 (14.77)	
40–44	600 (6.03)	2228 (7.3)	855 (6.7)	536 (6.42)	496 (7.53)	8925 (6.88)	
45–49	191 (1.92)	861 (2.82)	310 (2.43)	313 (3.75)	196 (2.97)	3118 (2.4)	
** Age at first birth (mean + S.E)**	18.61 ± 0.07	19.32 ± 0.07	18.04 ± 0.05	18.75 ± 0.06	20.33 ± 0.08	19.1 ± 0.02	
** Age at first birth group**							
< 15	1151 (11.55)	2469 (8.09)	1159 (9.08)	836 (10)	265 (4.03)	9770 (7.53)	
15–19	5374 (53.94)	15,784 (51.68)	8190 (64.15)	4557 (54.52)	2836 (43.1)	69,898 (53.89)	
20–24	2673 (26.83)	8599 (28.16)	2774 (21.73)	2305 (27.57)	2566 (38.99)	37,775 (29.13)	
25–29	595 (5.97)	2907 (9.52)	562 (4.4)	541 (6.47)	713 (10.83)	9882 (7.62)	
30–34	140 (1.41)	678 (2.22)	80 (0.62)	104 (1.24)	178 (2.71)	2032 (1.57)	
> 34	30 (0.31)	103 (0.34)	1 (0.01)	16 (0.19)	22 (0.34)	336 (0.26)	
** Highest educational level**							
No education	8283 (83.13)	15,093 (49.42)	10,963 (85.87)	5574 (66.69)	2681 (40.74)	82,958 (63.96)	
Primary	896 (8.99)	5879 (19.25)	1268 (9.93)	1167 (13.96)	2407 (36.57)	22,230 (17.14)	
Secondary	717 (7.2)	7826 (25.62)	497 (3.89)	1504 (17.99)	1392 (21.16)	21,390 (16.49)	
Higher	67 (0.67)	1742 (5.7)	39 (0.3)	114 (1.36)	101 (1.53)	3115 (2.4)	
** Current employment status**							
Not currently employed	5641 (56.62)	9500 (31.11)	9876 (77.36)	2064 (24.7)	1327 (20.17)	47,078 (36.3)	
Currently employed	4323 (43.38)	21,040 (68.89)	2891 (22.64)	6294 (75.3)	5253 (79.83)	82,615 (63.7)	
** Marital status**							
Not current married	251 (2.52)	1289 (4.22)	238 (1.86)	1313 (15.7)	476 (7.23)	9136 (7.04)	
Currently married/union	9712 (97.48)	29,251 (95.78)	12,529 (98.14)	7046 (84.3)	6105 (92.77)	120,557 (92.96)	
** Residence**							
Urban	1932 (19.39)	10,617 (34.76)	1630 (12.77)	2265 (27.1)	2363 (35.9)	40,351 (31.11)	
Rural	8032 (80.61)	19,924 (65.24)	11,137 (87.23)	6094 (72.9)	4218 (64.1)	89,342 (68.89)	
** Wealth**							
Poorest	2029 (20.36)	7248 (23.73)	2526 (19.79)	1850 (22.13)	1441 (21.9)	28,492 (21.97)	
Poorer	2075 (20.83)	7104 (23.26)	2597 (20.34)	1760 (21.06)	1316 (20)	28,092 (21.66)	
Middle	2053 (20.6)	5758 (18.85)	2671 (20.92)	1734 (20.74)	1304 (19.82)	26,043 (20.08)	
Richer	2106 (21.14)	5392 (17.66)	2749 (21.53)	1643 (19.65)	1284 (19.52)	25,378 (19.57)	
Richest	1700 (17.07)	5039 (16.5)	2224 (17.42)	1372 (16.42)	1234 (18.76)	21,688 (16.72)	
** Religion**							
Christian	416 (4.18)	11,185 (36.62)	–	1537 (18.39)	3429 (52.11)	40,835 (31.49)	
Muslim	9198 (92.32)	19,066 (62.43)	–	6797 (81.32)	1224 (18.6)	67,315 (51.9)	
No religion	243 (2.44)	–	–	4 (0.04)	622 (9.46)	3393 (2.62)	
Other religion	106 (1.07)	9 (0.03)	–	17 (0.21)	4 (0.06)	2001 (1.54)	
Tradition	–	281 (0.92)	–	3 (0.04)	1301 (19.77)	3381 (2.61)	
**Child characteristics**							
**Birth type**							
Singleton	9640 (96.75)	29,475 (96.51)	12,338 (96.64)	8172 (97.77)	6260 (95.13)	124,759 (96.2)	
Multiple	324 (3.25)	1065 (3.49)	429 (3.36)	186 (2.23)	320 (4.87)	4934 (3.8)	
**Sex of child**							
Male	5141 (51.59)	15,399 (50.42)	6448 (50.51)	4126 (49.36)	3320 (50.45)	65,826 (50.76)	
Female	4823 (48.41)	15,142 (49.58)	6319 (49.49)	4233 (50.64)	3261 (49.55)	63,867 (49.24)	
**Child size at birth**							
Large	4313 (43.29)	13,350 (43.71)	2374 (18.59)	3731 (44.63)	2228 (33.86)	49,601 (38.24)	
Average	4295 (43.11)	12,531 (41.03)	7059 (55.29)	3196 (38.24)	3274 (49.75)	58,713 (45.27)	
Small	1355 (13.6)	4659 (15.26)	3334 (26.11)	1432 (17.13)	1079 (16.39)	21,379 (16.48)	
**Birth order**							
1st-3rd	5172 (51.91)	15,716 (51.46)	5371 (42.07)	4444 (53.17)	3901 (59.29)	69,525 (53.61)	
4th–6th	3526 (35.39)	9705 (31.78)	4456 (34.9)	2783 (33.29)	2018 (30.66)	41,850 (32.27)	
7th + chi	1265 (12.7)	5120 (16.76)	2940 (23.03)	1131 (13.54)	662 (10.05)	18,318 (14.12)	
**Preceding birth interval**							
< 12 months	1948 (19.55)	6135 (20.09)	1932 (15.14)	1763 (21.09)	1584 (24.07)	26,925 (20.76)	
11 to 23 months	1563 (15.69)	5481 (17.95)	2397 (18.77)	896 (10.72)	638 (9.7)	17,449 (13.45)	
24 to 35 months	2887 (28.97)	9630 (31.53)	4724 (37)	2199 (26.31)	1597 (24.27)	38,118 (29.39)	
> 35 months	3566 (35.79)	9295 (30.43)	3714 (29.09)	3500 (41.88)	2761 (41.96)	47,201 (36.39)	
**Delivery type**							
Skilled delivery	5624 (56.44)	10,831 (35.46)	3801 (29.77)	4812 (57.57)	4800 (72.95)	70,529 (54.38)	
Unskilled delivery	4340 (43.56)	19,709 (64.54)	8966 (70.23)	3547 (42.43)	1780 (27.05)	59,163 (45.62)	
**Delivery mode**							
SVD	9690 (97.25)	29,910 (97.93)	12,584 (98.56)	8016 (95.91)	6144 (93.36)	125,350 (96.65)	
Caesarea	274 (2.75)	631 (2.07)	183 (1.44)	342 (4.09)	437 (6.64)	4343 (3.35)	
**Death status**							
Alive	9268 (93.02)	27,898 (91.35)	11,738 (91.94)	7703 (92.16)	6206 (94.31)	120,228 (92.7)	
Dead	695 (6.98)	2643 (8.65)	1029 (8.06)	656 (7.84)	375 (5.69)	9465 (7.3)	

*S.E* standard error, *SVD* spontaneous vagina delivery

Majority of the mothers had no formal education. Niger (85.8%), Burkina Faso (84.3%) and Mali (83.1%) recorded the highest percentage whilst Nigeria (49.4%), Liberia (41.7%) and Ghana (27.4%) recorded the least percentage of women with no formal education. Less than a fifth of them obtained primary or secondary level of education and a very few of them had attained higher level of education, Nigeria (5.7%) and Ghana (4.4) compared to the other West African countries in the study.

More than half (68.9%) of the respondents in the West African countries resided in rural areas. Similar pattern prevailed within the individual countries. Majority (63.7%) of the women were currently employed with a little over a third (36.3%) of them not currently employed. The employment distribution was similar within the countries except for Gambia (52.1%), Mali (56.6%), and Niger (77.4%) where majority of the women were currently unemployed. Nine out of ten (93.0%) of the women were married or in a union. Similar situation existed within the countries except for Cote d’lvoire (84.9%), Ghana (85.6%), Liberia (75.6%) and Sierra Leone (84.3%) where less than nine out of ten of the mothers were currently married or in a union.

Muslims were the majority group in the study in all twelve countries followed by Christians. Within country distribution, Muslims were the majority group in Gambia (97.6%), Sierra Leone (92.7%), Mali (92.3%), Guinea (87.8%), Burkina (63.8%), and Nigeria (62.4%) whilst Christians were the majority in Liberia (84.4%), Ghana (75.6%), Benin (56.2%) and then Togo (52.1%).

In terms of the child characteristics, the sample was approximately equally distributed among the males and females in all the countries. Most of the children had an average birth weight (45.27%) with less than 20% of them having small birth weight. There was however disparity between the countries as Cote D’lvoire (49.7%), Ghana (51.1%), Gambia (50.8%), Guinea (48.3%), Liberia (46.2%), Mali (43.3%), Nigeria (43.7%), and Sierra Leone (44.6%) had most of the children born with large body weight. Majority of the children were first to third born of their mothers with less than a fifth of them being at least the 7th born of their mothers. Majority of the children were delivered by a skilled birth attendant. Niger (29.8%) was the only country which had minority of the children delivered by skilled birth attendants. Less than 5% of the children were birthed through caesarean section, which was a pattern throughout the West African countries except for Benin and Togo which had 5.6 and 6.6% of the children being born by caesarian section respectively, Table 1.

### Under-five mortality rates per 1000 livebirths

**Fig. 1:**
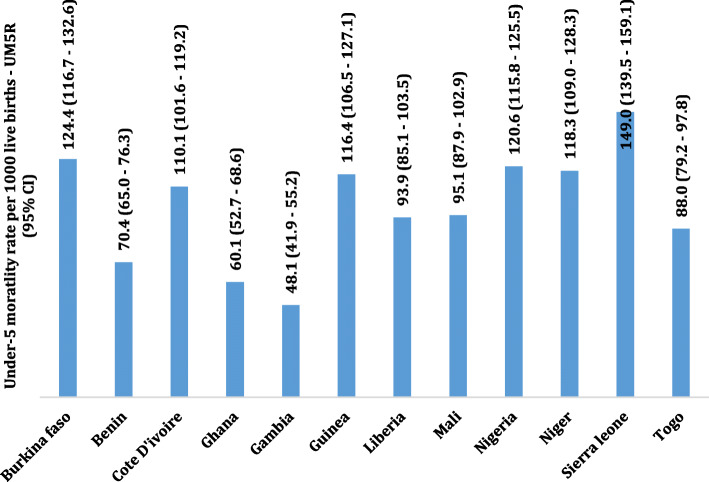
Under-5 mortality in the West African countries

**Fig. 2:**
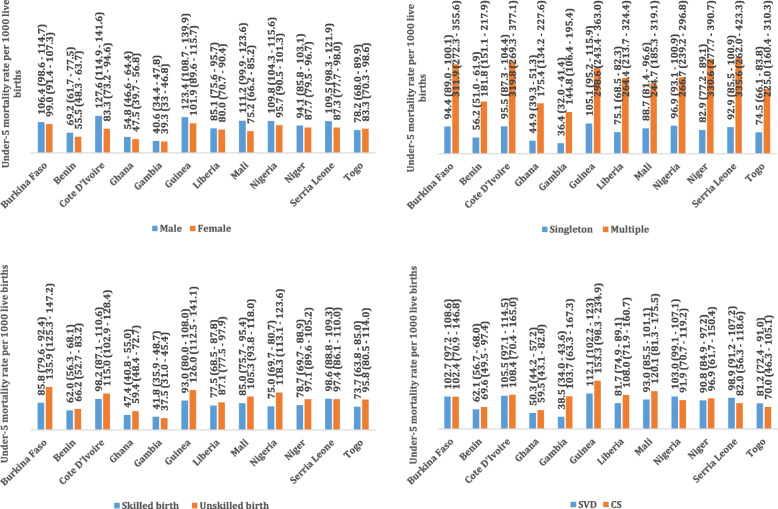
Under-five mortality rates by child sex, type of birth of child, delivery type of child and mode of delivery of the child for all the 12 West African countries with each of the country specific mortality rate

**Fig. 3:**
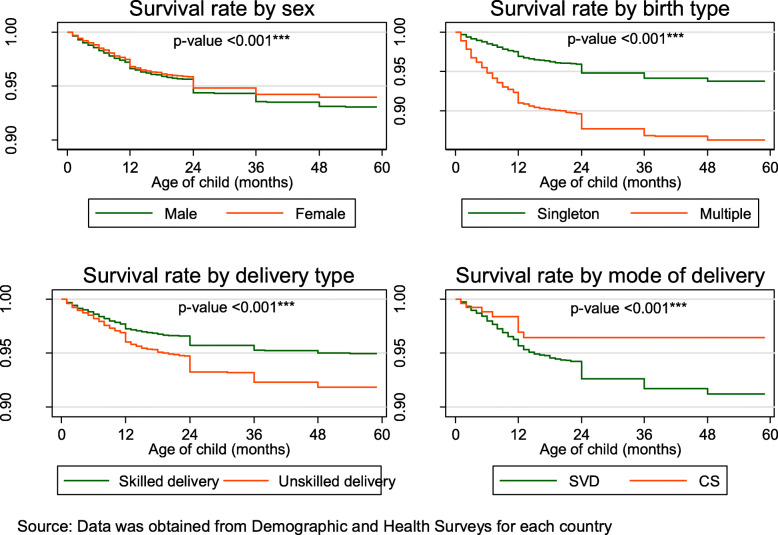
Kaplan-Meier survival curves for sex of the child, birth type, delivery type and mode of delivery for the combined data set across all the 12 West African countries with their *p*-values obtained from the loglank test

**Fig. 4:**
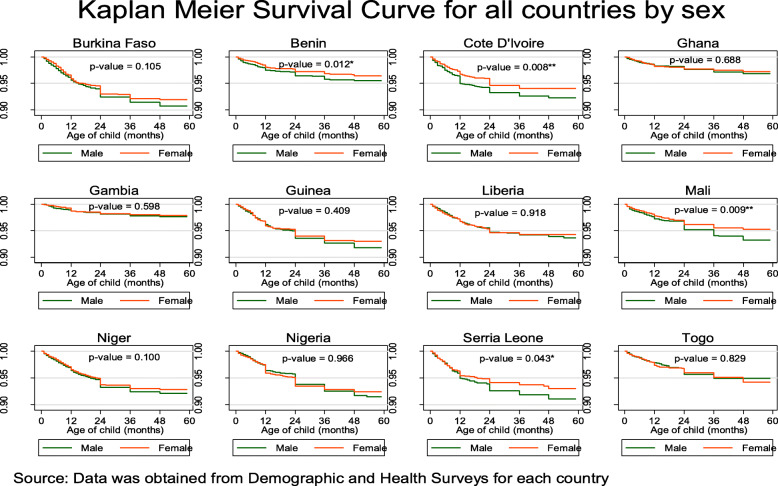
Kaplan-Meier survival curves for sex of the child across all the 12 West African countries with each of the country specific data

**Fig. 5:**
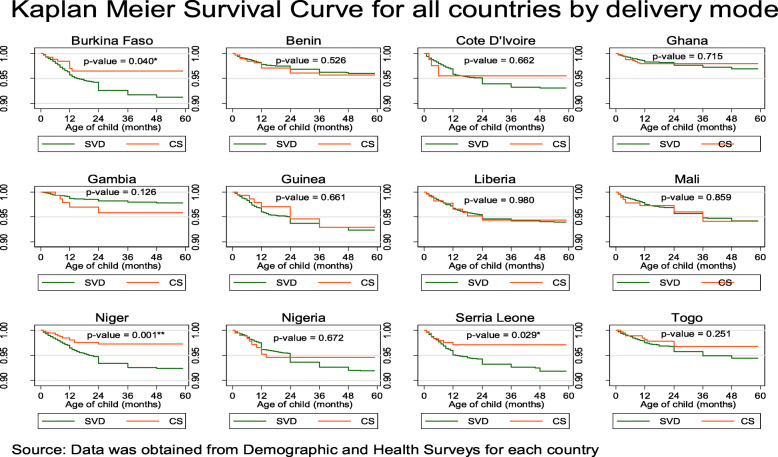
Kaplan-Meier survival curves for mode of delivery of the child across all the 12 West African countries with each of the country specific data

**Fig. 6:**
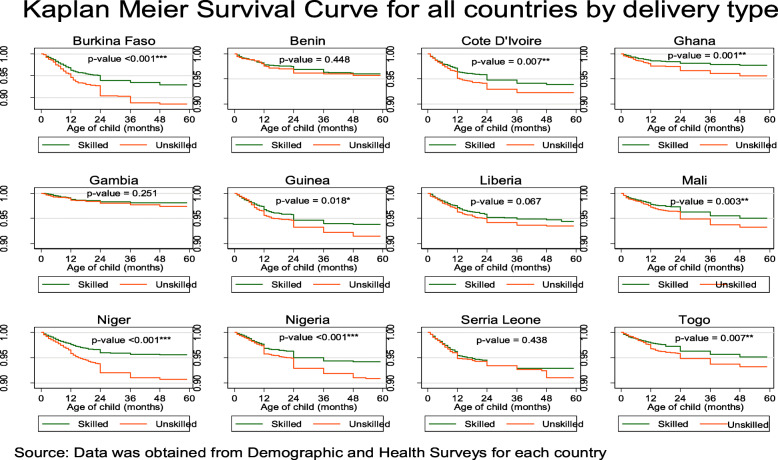
Kaplan-Meier survival curves for place of delivery of the child across all the 12 West African countries with each of the country specific data

**Fig. 7:**
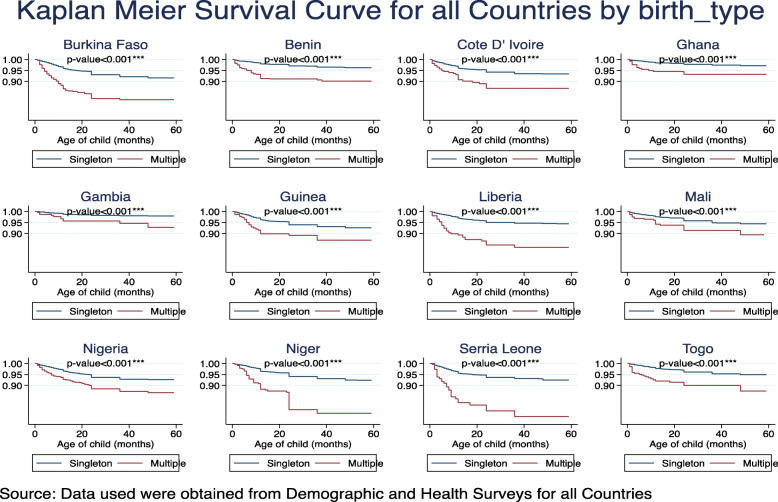
Kaplan-Meier survival curves for birth type of the child across all the 12 West African countries with each of the country specific data

Among the 129,693 whose samples were included in this study, 101 with a 95% CI of (98.6–103.5) deaths per 1000 live births occurred among the twelve countries in the last 5 years preceding the survey. Burkina Faso 124.4, Cote D’lvoire 110.1, Guinea 116.4, Nigeria 120.6 and Niger 118.3 recorded the highest child under-5 mortality rate all of which were above 100 death per 1000 livebirths in the 5 years preceding the survey. Gambia 48.1, Ghana 60.1 and Benin 70.4 recorded the least under-5 mortality rate per 1000 livebirths in the 5 years preceding the survey, Table 1. The overall country specific under-five mortality rate per 1000 livebirths has been provided in Fig. 1. Further to this is also the under-five mortality rates per 1000 livebirths for four most important variables used to predict child mortality, namely; sex of the child, mode of delivery of the child, delivery type and birth type, Fig. 2. Figure 3, presents the Kaplan-Meier survival functions of the combined dataset for under-five mortality according to sex, mode of delivery, delivery type and birth type while Figs. 4, 5, 6, 7 are the country specific estimates. A statistically significant difference between all the variables were observed. The survival functions show that, caesarian section, unskilled delivery, males and multiple births are all significantly higher than normal, skilled delivery, females and singleton births.

### Model evaluation and cross validation checks

The best model was determined using deviance information criteria and Bayes factors as presented below

**Table:** 

Models	DIC	BF
M1	58,449.67	–
M2	57,975.33	261.74
M3	57,183.99	578.34
M4	58,440.43	29.02
M5	57,977.01	299.73
M6	57,146.23	641.48

Model M1 represents the exponential regression model without frailty, M2 is the Weibull regression model without frailty whilst M3 is the Gompertz regression model without frailty. The remaining M4, M5 and M6 represent the exponential, Weibull and Gompertz regression models with gamma shared frailty terms. Observing from the table above, model M1 is the worst performing model compared to all the others including the exponential model with gamma shared frailty. This is because it has the highest DIC value. Though Weibull performs well, Gompertz is the best performing model whether with or without the frailty term. We can therefore conclude that the strongest and decisive model is the Gompertz model with a gamma shared frailty. This model was further subjected to Bayesian goodness of fit test using posterior probabilities (PP). The PP value obtained was 1.00, which indicates strongly that the Gompertz model with a gamma shared frailty was a good fit to the data. Therefore, the final analysis and results interpretation were based on the Gompertz regression model with a frailty term.

### Predictors of under-5 mortality in West Africa

Table 2, shows the hazard rates of under-5 mortality in the twelve West African countries considered in study with a Bayesian Gompertz’s regression modeling approach via a gamma shared frailty. In all the countries analyses, there were differentials in the under-5 mortality rates for all the socio-demographic characteristics of both mother and child observed in the study except for the delivery mode of the child. Togo (HR: 0.92, 95% CI: [0.79–1.03]) was the only country that did not have any significant difference in the hazard rate of the under-5 mortality when compared to Ghana after all the observed factors in the study were adjusted for. Compared to Ghana, the adjusted hazard rate of under-5 mortality was significantly lower in Benin (HR: 0.67, 95% CI: [0.64–0.70]), Cote D’lvoire (HR: 0.75, 95% CI: [0.70–0.79]), Gambia (HR: 0.74, 95% CI: [0.67–0.80]) and Guinea (HR: 0.84, 95% CI: [0.74–0.93]), whilst it was significantly higher in Burkina Faso (HR: 1.65, 95% CI: [1.56–1.74]), Liberia (HR: 1.47, 95% CI: [1.36–1.58]), Mali (HR: 1.19, 95% CI: [1.06–1.33]), Nigeria (HR: 1.65, 95% CI: [1.54–1.77]), Niger (HR: 1.51: 95% CI: [1.38–1.64]) and Sierra Leone (HR: 1.86, 95% CI: [1.69–2.03]).

**Table 2:** Predictors of under-5 mortality in West Africa, namely; Ghana, Benin, Cote d’ Ivoire, Guinea, Liberia, Mali, Niger, Nigeria, Sierra Leone, Burkina Faso, Gambia and Togo

	**Burkina Faso**	**Benin**	**Cote D’lvoire**	**Ghana**	**Gambia**	**Guinea**	**Liberia**
**Characteristics**	**HR [95% CI]**	**HR [95% CI]**	**HR [95% CI]**	**HR [95% CI]**	**HR [95% CI]**	**HR [95% CI]**	**HR [95% CI]**
**Mothers’ characteristics**							
**Mother’s current age**							
15–19	1.82 [1.28–2.23]*	1.08 [0.86–1.33]	1.23 [1.07–1.41]*	0.99 [0.70–1.29]	4.51 [3.28–6.03]*	1.78 [1.29–2.4]*	1.24 [0.94–1.59]
20–24	1.00 [0.77–1.28]	1.86 [1.57–2.18]*	0.6 [0.51–0.69]*	1.18 [0.90–1.5]	1.44 [1.11–1.79]*	1.12 [1.02–1.26]*	0.99 [0.77–1.25]
25–29	1.20 [0.94–1.50]	1.23 [1.06–1.42]*	0.74 [0.65–0.84]*	1.30 [0.96–1.72]	1.52 [1.22–1.84]*	1.26 [1.11–1.44]*	0.90 [0.78–1.03]
30–34	0.98 [0.76–1.17]	1.47 [1.21–1.75]*	0.85 [0.70–1.01]	1.72 [1.44–2.01]*	0.81 [0.58–1.07]	0.63 [0.55–0.73]*	0.94 [0.83–1.05]
35–39	1.07 [0.80–1.34]	1.01 [0.84–1.22]	1.49 [1.28–1.73]*	0.80 [0.53–1.14]	0.81 [0.64–1.03]	0.57 [0.46–0.70]*	0.56 [0.44–0.69]*
40–44	0.88 [0.70–1.09]	1.63 [1.34–1.97]*	0.93 [0.71–1.18]	0.88 [0.58–1.28]	2.20 [1.66–2.84]*	0.72 [0.65–0.79]*	0.60 [0.53–0.68]*
45–49	1.00 [reference]	1.00 [reference]	1.00 [reference]	1.00 [reference]	1.00 [reference]	1.00 [reference]	1.00 [reference]
**Mother’s age at first birth**							
< 15 years	1.00 [reference]	1.00 [reference]	1.00 [reference]	1.00 [reference]	1.00 [reference]	1.00 [reference]	1.00 [reference]
15–19 years	1.00 [0.74–1.43]	1.52 [1.37–1.69]*	1.04 [0.88–1.21]	1.19 [0.92–1.57]	0.32 [0.27–0.37]*	1.02 [0.86–1.20]	1.18 [0.99–1.37]
20–24 years	1.00 [0.76–1.27]	1.70 [1.41–2.03]*	0.89 [0.75–1.04]	1.47 [1.23–1.76]*	0.19 [0.14–0.27]*	1.50 [1.26–1.78]*	1.3 [1.08–1.54]*
25–29 years	1.06 [0.71–1.71]	1.31 [1.13–1.51]*	0.51 [0.44–0.59]*	1.52 [1.10–2.00]*	0.36 [0.26–0.49]*	1.79 [1.37–2.23]*	0.93 [0.76–1.14]
30–34 years	0.68 [0.20–1.49]	0.33 [0.26–0.41]*	1.11 [0.85–1.41]	1.45 [1.12–1.84]*	0.62 [0.47–0.82]*	0.42 [0.36–0.50]*	1.00 [0.79–1.25]
> 34 years	1.15 [0.55–2.44]	0.78 [0.65–0.92]*	1.53 [1.25–1.84]*	1.64 [1.13–2.32]*	1.33 [0.97–1.81]	0.53 [0.44–0.62]*	0.45 [0.35–0.58]*
**Highest level of education**							
No education	1.00 [reference]	1.00 [reference]	1.00 [reference]	1.00 [reference]	1.00 [reference]	1.00 [reference]	1.00 [reference]
Primary	0.81 [0.71–0.92]	0.85 [0.73–0.97]*	1.18 [1.01–1.37]*	0.91 [0.73–1.13]	1.26 [0.86–1.76]	1.02 [0.88–1.17]	0.77 [0.63–0.94]*
Secondary	0.84 [0.76–0.92]	0.86 [0.72–1.01]	0.64 [0.46–0.86]*	0.66 [0.51–0.84]*	1.07 [0.701–1.51]	0.67 [0.56–0.80]*	0.80 [0.67–0.96]*
Tertiary	1.64 [1.33–2.03] *	0.89 [0.72–1.07]	0.52 [0.37–0.72]*	1.43 [0.89–2.13]	2.40 [1.27–4.18]*	0.90 [0.71–1.15]	1.16 [0.79–1.68]
**Current employment status**							
Currently employed	1.00 [reference]	1.00 [reference]	1.00 [reference]	1.00 [reference]	1.00 [reference]	1.00 [reference]	1.00 [reference]
Currently unemployed	1.22 [1.02–1.43]*	1.21 [1.03–1.39]*	0.93 [0.82–1.06]	0.96 [0.76–1.21]	0.78 [0.63–0.97]*	0.59 [0.52–0.67]*	1.11 [1.04–1.18]*
**Marital status**							
Not currently married	1.00 [reference]	1.00 [reference]	1.00 [reference]	1.00 [reference]	1.00 [reference]	1.00 [reference]	1.00 [reference]
Currently married/union	0.65 [0.48–0.84]*	0.65 [0.56–0.75]*	0.99 [0.86–1.14]	0.52 [0.35–0.71]*	0.63 [0.43–0.93]*	0.49 [0.4–0.61]*	0.62 [0.53–0.71]*
**Residence**							
urban	1.00 [reference]	1.00 [reference]	1.00 [reference]	1.00 [reference]	1.00 [reference]	1.00 [reference]	1.00 [reference]
Rural	1.3 [1.06–1.61]*	1.82 [1.57–2.09]*	1.44 [1.21–1.68]*	1.05 [0.84–1.32]	0.16 [0.12–0.2]*	0.62 [0.51–0.73]*	0.96 [0.78–1.18]
**Wealth**							
Poorest	1.53 [1.23–1.8]*	0.91 [0.79–1.08]	1.17 [0.96–1.4]	0.86 [0.64–1.12]	0.99 [0.73–1.29]	1.09 [0.97–1.23]	1.08 [0.94–1.24]
Poorer	1.62 [1.35–1.99]*	0.83 [0.73–0.94]*	0.77 [0.66–0.89]*	0.99 [0.72–1.3]	0.54 [0.36–0.74]*	1.16 [1.01–1.32]*	1.3 [1.14–1.49]*
Middle	1.38 [1.12–1.71]*	0.97 [0.84–1.08]	1.04 [0.87–1.24]	1.23 [0.95–1.58]	0.79 [0.53–1.11]	1.18 [1.00–1.37]	1.49 [1.25–1.72]*
Richer	1.38 [1.14–1.71]*	0.69 [0.6–0.78]*	1.14 [0.97–1.32]	1.32 [1.08–1.6]*	0.51 [0.39–0.65]*	0.45 [0.37–0.52]*	1.05 [0.90–1.22]
Richest	1.00 [reference]	1.00 [reference]	1.00 [reference]	1.00 [reference]	1.00 [reference]	1.00 [reference]	1.00 [reference]
**Religion**							
Christian	1.00 [reference]	1.00 [reference]	1.00 [reference]	1.00 [reference]	1.00 [reference]	1.00 [reference]	1.00 [reference]
Muslim	1.12 [0.96–1.29]	0.78 [0.68–0.90]*	1.19 [1.02–1.38]*	1.45 [1.10–1.87]*	0.13 [0.11–0.17]*	1.01 [0.87–1.18]	1.71 [1.39–2.09]*
No religion	1.17 [0.56–1.90]	0.58 [0.52–0.65]*	0.96 [0.79–1.14]	1.13 [0.90–1.45]	1.84 [1.49–2.3]*	2.50 [1.90–3.19]*	0.99 [0.78–1.23]
Other religion	0.48 [0.19–1.04]	1.45 [1.17–1.79]*	1.02 [0.9–1.15]	–	–	5.76 [4.74–6.86]*	1.05 [0.77–1.41]
Traditional	1.49 [1.18–1.88]*	1.18 [0.57–2.05]	–	1.71 [1.15–2.51]*	–	–	0.01 [0.00–0.02]*
**Child characteristics**							
**Birth type**							
Singleton	1.00 [reference]	1.00 [reference]	1.00 [reference]	1.00 [reference]	1.00 [reference]	1.00 [reference]	1.00 [reference]
Multiple	2.83 [2.41–3.19]*	2.47 [2.09–2.87]*	1.79 [1.45–2.19]*	2.29 [1.68–3.07]*	1.22 [0.89–1.59]	1.89 [1.49–2.38]*	2.23 [1.73–2.8]*
**Sex**							
Female	1.00 [reference]	1.00 [reference]	1.00 [reference]	1.00 [reference]	1.00 [reference]	1.00 [reference]	1.00 [reference]
Male	1.1 [0.95–1.24]	1.36 [1.2–1.54]*	1.18 [1.04–1.34]*	1.2 [0.97–1.49]	0.84 [0.63–1.09]	1.22 [1.07–1.39]*	1.16 [1.01–1.31]*
**Child size at birth**							
Large	1.00 [reference]	1.00 [reference]	1.00 [reference]	1.00 [reference]	1.00 [reference]	1.00 [reference]	1.00 [reference]
Average	1.26 [1.00–1.49]	0.94 [0.81–1.08]	1.10 [0.92–1.29]	0.80 [0.67–0.94]*	0.76 [0.58–0.96]*	1.28 [1.06–1.5]*	0.96 [0.79–1.16]
Small	1.37 [0.98–1.71]	1.66 [1.39–1.95]*	0.98 [0.87–1.09]	1.63 [1.33–1.96]*	0.71 [0.57–0.87]*	1.49 [1.23–1.79]*	1.32 [1.09–1.57]*
**Birth order**							
1st-3rd child	1.00 [reference]	1.00 [reference]	1.00 [reference]	1.00 [reference]	1.00 [reference]	1.00 [reference]	1.00 [reference]
4th–6th child	0.99 [0.75–1.22]	1.39 [1.18–1.62]*	0.76 [0.66–0.88]*	1.30 [1.07–1.58]*	0.86 [0.65–1.13]	1.46 [1.3–1.64]*	1.07 [0.9–1.28]
7th + child	1.21 [0.86–1.66]	2.41 [2.19–2.65]*	0.51 [0.44–0.59]*	1.85 [1.3–2.56]*	0.68 [0.53–0.86]*	1.41 [1.23–1.61]*	0.93 [0.82–1.04]
**Preceding birth interval**							
< 12 months	1.46 [1.18–1.79]*	0.94 [0.84–1.06]	1.07 [0.89–1.26]	0.98 [0.79–1.19]	0.60 [0.49–0.73]*	0.81 [0.74–0.89]*	1.24 [1.1–1.41]*
11 to 23 months	2.35 [1.95–2.83]*	1.81 [1.5–2.16]*	1.86 [1.62–2.12]*	1.08 [0.89–1.33]	1.56 [1.35–1.79]*	1.29 [1.09–1.53]*	1.73 [1.41–2.1]*
24 to 35 months	1.81 [1.56–2.10]*	1.12 [0.98–1.28]	1.13 [1.01–1.27]*	1.01 [0.74–1.34]	0.65 [0.52–0.82]*	1.26 [1.10–1.44]*	1.3 [1.10–1.53]*
> 35 months	1.00 [reference]	1.00 [reference]	1.00 [reference]	1.00 [reference]	1.00 [reference]	1.00 [reference]	1.00 [reference]
**Type of delivery**							
Skilled	1.00 [reference]	1.00 [reference]	1.00 [reference]	1.00 [reference]	1.00 [reference]	1.00 [reference]	1.00 [reference]
Unskilled	1.20 [1.03–1.38]*	0.88 [0.68–1.09]	1.20 [1.05–1.39]*	1.59 [1.29–1.9]*	1.73 [1.26–2.32]*	1.52 [1.33–1.71]*	1.32 [1.18–1.46]*
**Delivery mode**							
SVD	1.00 [reference]	1.00 [reference]	1.00 [reference]	1.00 [reference]	1.00 [reference]	1.00 [reference]	1.00 [reference]
Caesarean	0.68 [0.5–0.94]*	0.96 [0.71–1.23]	0.91 [0.71–1.13]	1.49 [1.17–1.84]*	1.66 [1.3–2.08]*	1.96 [1.64–2.32]*	0.77 [0.62–0.92]*
**Country**							
Ghana	–	–	–	–	–	–	–
Burkina Faso	–	–	–	–	–	–	–
Benin	–	–	–	–	–	–	–
Cote D’Ivoire	–	–	–	–	–	–	–
Gambia	–	–	–	–	–	–	–
Guinea	–	–	–	–	–	–	–
Liberia	–	–	–	–	–	–	–
Mali	–	–	–	–	–	–	–
Nigeria	–	–	–	–	–	–	–
Niger	–	–	–	–	–	–	–
Serria Leone	–	–	–	–	–	–	–
Togo	–	–	–	–	–	–	–
**Gamma**	−0.04 [−0.04–0.03]*	−0.05 [−0.06--0.04]*	−0.05 [−0.06--0.04]*	−0.05 [− 0.07--0.04]*	−0.07 [− 0.09--0.05]*	−0.04 [− 0.05--0.03]*	−0.05 [− 0.06--0.04]*
**ln (theta)**	−7.75 [−47.5–1.65]*	−0.41 [− 0.96–0.04]*	−1.2 [−1.98--0.62]*	− 70.28 [− 223.76--0.54]*	0.8 [0.13–1.4]	− 0.94 [− 1.65--0.39]*	−63.19 [− 209.6--1.23]*
	**Mali**	**Nigeria**	**Niger**	**Serria Leone**	**Togo**	**Overall**	
**Characteristics**	**HR [95% CI]**	**HR [95% CI]**	**HR [95% CI]**	**HR [95% CI]**	**HR [95% CI]**	**HR [95% CI]**	
**Mothers’ characteristics**							
** Mother’s current age**							
15–19	1.00 [reference]	1.00 [reference]	1.00 [reference]	1.00 [reference]	1.00 [reference]	1.00 [reference]	
20–24	1.23 [0.98–1.68]	0.69 [0.65–0.75]*	0.45 [0.37–0.54]*	1.95 [1.58–2.4]*	1.93 [1.29–2.74]*	1.59 [1.54–1.64]*	
25–29	1.25 [0.93–1.6]	0.73 [0.67–0.8]*	0.59 [0.52–0.67]*	1.37 [1.15–1.59]*	1.06 [0.85–1.31]	1.43 [1.37–1.49]*	
30–34	1.19 [0.94–1.51]	0.67 [0.63–0.71]*	0.50 [0.45–0.57]*	1.14 [0.89–1.46]	0.79 [0.66–0.93]*	1.32 [1.27–1.4]*	
35–39	1.2 0 [0.93–1.60]	0.83 [0.76–0.90]*	0.50 [0.43–0.58]*	0.99 [0.80–1.20]	0.84 [0.72–0.95]*	1.11 [1.09–1.15]*	
40–44	1.34 [0.92–1.84]	1.14 [1.08–1.20]*	0.63 [0.55–0.71]*	1.40 [1.18–1.71]*	0.79 [0.65–0.95]*	1.09 [1.05–1.13]*	
45–49	2.15 [1.7–2.63]*	0.80 [0.72–0.88]*	0.43 [0.36–0.51]*	0.96 [0.76–1.20]	1.02 [0.74–1.39]	1.08 [1.02–1.18]*	
**Mother’s age at first birth**							
< 15 years	1.00 [reference]	1.00 [reference]	1.00 [reference]	1.00 [reference]	1.00 [reference]	1.00 [reference]	
15–19 years	0.91 [0.75–1.12]	0.92 [0.88–0.96]*	0.98 [0.84–1.14]	0.93 [0.8–1.09]	0.69 [0.54–0.87]*	1.2 [1.18–1.22]*	
20–24 years	0.84 [0.68–1.03]	0.90 [0.84–0.96]*	0.89 [0.75–1.04]	1.05 [0.88–1.24]	0.73 [0.63–0.84]*	1.15 [1.11–1.18]*	
25–29 years	0.84 [0.63–1.12]	0.76 [0.71–0.81]*	0.74 [0.64–0.84]*	0.91 [0.75–1.1]	0.47 [0.33–0.64]*	1.28 [1.20–1.36]*	
30–34 years	1.17 [0.84–1.67]	0.96 [0.91–1.02]	1.06 [0.97–1.15]	0.84 [0.69–1.01]	0.76 [0.6–0.98]*	1.62 [1.44–1.84]*	
> 34 years	1.68 [1.35–2.16]*	0.99 [0.92–1.06]	0.83 [0.72–0.95]*	1.04 [0.86–1.22]	2.48 [1.79–3.22]*	2.52 [2.43–2.64]*	
**Highest level of education**							
No education	1.00 [reference]	1.00 [reference]	1.00 [reference]	1.00 [reference]	1.00 [reference]	1.00 [reference]	
Primary	1.21 [1.06–1.40]*	0.97 [0.88–1.07]	0.85 [0.71–1.12]	1.22 [1.02–1.48]*	1.13 [0.97–1.33]	0.91 [0.89–0.94]*	
Secondary	0.96 [0.69–1.29]	0.85 [0.75–0.95]*	0.94 [0.71–1.20]	1.35 [1.24–1.48]*	0.75 [0.61–0.93]	0.73 [0.69–0.76]*	
Tertiary	0.99 [0.84–1.16]	0.84 [0.78–0.92]*	0.53 [0.34–0.78]*	0.61 [0.38–0.89]*	0.98 [0.77–1.23]	0.82 [0.78–0.86]*	
** Current employment status**							
Currently employed	1.00 [reference]	1.00 [reference]	1.00 [reference]	1.00 [reference]	1.00 [reference]	1.00 [reference]	
Currently unemployed	0.74 [0.61–0.92]*	0.94 [0.86–1.03]	0.96 [0.82–1.11]	0.59 [0.48–0.74]*	1.16 [0.91–1.47]	0.90 [0.84–0.95]*	
**Marital status**							
Not currently married	1.00 [reference]	1.00 [reference]	1.00 [reference]	1.00 [reference]	1.00 [reference]	1. [reference]	
Currently married/union	0.47 [0.37–0.59]*	0.94 [0.9–0.98]*	0.3 [0.24–0.37]*	0.68 [0.54–0.86]*	1.46 [1.09–1.89]*		
**Residence**							
Urban	1.00 [reference]	1.00 [reference]	1.00 [reference]	1.00 [reference]	1.00 [reference]	1.00 [reference]	
Rural	1.77 [1.41–2.14]*	1.34 [1.23–1.46]*	5 [4.49–5.52]*	1.2 [0.94–1.46]	0.86 [0.71–1.03]	1.45 [1.37–1.55]*	
**Wealth**							
Poorest	1.80 [1.44–2.25]*	0.63 [0.6–0.66]*	0.85 [0.73–0.98]*	0.76 [0.61–0.94]*	1.61 [1.47–1.77]*	0.78 [0.74–0.82]*	
Poorer	1.21 [0.94–1.52]	0.67 [0.64–0.71]*	1.08 [0.93–1.25]	0.97 [0.75–1.26]	1.5 [1.27–1.74]*	0.93 [0.91–0.96]*	
Middle	1.61 [1.37–1.88]*	0.64 [0.59–0.7]*	1.00 [0.83–1.20]	0.94 [0.75–1.16]	0.82 [0.67–0.99]*	0.87 [0.82–0.92]*	
Richer	1.14 [0.96–1.39]	0.67 [0.64–0.71]*	1.02 [0.88–1.16]	0.95 [0.76–1.15]	0.81 [0.62–1.06]	0.87 [0.83–0.90]*	
Richest	1.00 [reference]	1.00 [reference]	1.00 [reference]	1.00 [reference]	1.00 [reference]	1.00 [reference]	
**Religion**							
Christian	1.00 [reference]	1.00 [reference]	–	1.00 [reference]	1.00 [reference]	1.00 [reference]	
Muslim	0.79 [0.55–1.08]	0.88 [0.81–0.96]*	–	0.97 [0.83–1.12]	1.14 [0.94–1.32]	1.25 [1.17–1.35]*	
No religion	1.28 [0.97–1.89]	–	–	0.36 [0.25–0.52]*	0.69 [0.59–0.79]*	2.22 [2.08–2.37]*	
Other religion	0.94 [0.69–1.18]	0.67 [0.63–0.72]*	–	1.77 [1.3–2.28]*	1.43 [1.09–1.87]*	2.01 [1.68–2.45]*	
Traditional	–	0.43 [0.39–0.47]*	–	0.04 [0.00–0.18]*	1.07 [0.78–1.43]	1.39 [1.26–1.54]*	
**Child characteristics**							
**Birth type**							
Singleton	1.00 [reference]	1.00 [reference]	1.00 [reference]	1.00 [reference]	1.00 [reference]	1.00 [reference]	
Multiple	2.26 [1.80–2.83]*	1.03 [0.96–1.10]	2.93 [2.46–3.49]*	0.93 [0.79–1.08]	1.51 [1.15–1.93]*	2.81 [2.60–3.02]*	
**Sex**							
Female	1.00 [reference]	1.00 [reference]	1.00 [reference]	1.00 [reference]	1.00 [reference]	1.00 [reference]	
Male	1.25 [1.04–1.47]*	1.1 [1.05–1.16]*	0.88 [0.81–0.94]*	1.12 [0.94–1.31]	1.01 [0.85–1.19]	1.14 [1.10–1.18]*	
**Child size at birth**							
Large	1.00 [reference]	1.00 [reference]	1.00 [reference]	1.00 [reference]	1.00 [reference]	1.00 [reference]	
Average	1.11 [0.95–1.34]	1.13 [1.06–1.21]*	1.02 [0.91–1.15]	0.99 [0.86–1.15]	1.15 [0.94–1.37]	1.12 [1.06–1.17]*	
Small	1.43 [1.14–1.79]*	1.43 [1.35–1.52]*	1.03 [0.9–1.17]	1.53 [1.26–1.82]*	1.6 [1.29–1.93]*	1.48 [1.43–1.52]*	
**Birth order**							
1st-3rd child	1.00 [reference]	1.00 [reference]	1.00 [reference]	1.00 [reference]	1.00 [reference]	1.00 [reference]	
4th–6th child	0.91 [0.78–1.05]	0.82 [0.75–0.88]*	0.96 [0.82–1.12]	1.09 [0.85–1.35]	1.12 [0.90–1.39]	1.32 [1.28–1.36]*	
7th + child	1.12 [0.95–1.28]	0.78 [0.73–0.83]*	0.82 [0.7–0.95]*	1.74 [1.44–2.08]*	0.72 [0.58–0.89]*	1.62 [1.52–1.70]*	
**Preceding birth interval**							
< 12 months	2.42 [1.82–3.40]*	1.21 [1.1–1.32]*	1.24 [1.10–1.40]*	1.37 [1.08–1.72]*	0.78 [0.66–0.93]*	1.58 [1.49–1.66]*	
11 to 23 months	2.64 [2.03–3.40]*	1.88 [1.69–2.09]*	1.15 [0.99–1.33]	2.15 [1.69–2.66]*	1.97 [1.59–2.43]*	2.01 [1.90–2.11]*	
24 to 35 months	1.85 [1.45–2.31]*	1.26 [1.15–1.38]*	0.99 [0.84–1.15]	1.40 [1.22–1.60]*	1.65 [1.42–1.91]*	1.39 [1.34–1.44]*	
> 35 months	1.00 [reference]	1.00 [reference]	1.00 [reference]	1.00 [reference]	1.00 [reference]	1.00 [reference]	
**Type of delivery**							
Skilled	1.00 [reference]	1.00 [reference]	1.00 [reference]	1.00 [reference]	1.00 [reference]	1.00 [reference]	
Unskilled	0.84 [0.68–1.00]	1.25 [1.15–1.36]*	0.80 [0.71–0.90]*	0.92 [0.78–1.08]	1.24 [1.01–1.54]*	1.15 [1.08–1.22]*	
**Delivery mode**							
SVD	1.00 [reference]	1.00 [reference]	1.00 [reference]	1.00 [reference]	1.00 [reference]	1.00 [reference]	
Caesarean	1.24 [0.92–1.61]	0.25 [0.22–0.28]*	1.11 [0.91–1.34]	0.68 [0.54–0.83]*	0.76 [0.59–0.98]*	1.04 [0.94–1.14]	
**Country**							
Ghana	–	–	–	–	–	1.00 [reference]	
Burkina Faso	–	–	–	–	–	1.65 [1.56–1.74]*	
Benin	–	–	–	–	–	0.67 [0.64–0.7]*	
Cote D’Ivoire	–	–	–	–	–	0.75 [0.7–0.79]*	
Gambia	–	–	–	–	–	0.74 [0.67–0.8]*	
Guinea	–	–	–	–	–	0.84 [0.74–0.93]*	
Liberia	–	–	–	–	–	1.47 [1.36–1.58]*	
Mali	–	–	–	–	–	1.19 [1.06–1.33]*	
Nigeria	–	–	–	–	–	1.65 [1.54–1.77]*	
Niger	–	–	–	–	–	1.51 [1.38–1.64]*	
Serria Leone	–	–	–	–	–	1.86 [1.69–2.03]*	
Togo	–	–	–	–	–	0.92 [0.79–1.03]	
**Gamma**	−0.03 [−0.04--0.02]*	−0.04 [−0.04−−0.03]*	-0.03 [− 0.03--0.02]*	− 0.05 [− 0.06--0.04]*	−0.03 [− 0.04--0.02]*	−0.04 [− 0.04--0.03]*	
**ln (theta)**	−1.07 [−1.91--0.49]*	−1.47 [−1.85--1.16]*	−0.88 [− 1.34--0.5]*	−79.42 [− 220.4--3.38]*	−74.32 [−228.84--1.29]*	−1.17 [− 1.34--1]*	

*CI* credible interval, *HR* Hazard rate. *: *p*-value < 0.05. *SVD* Spontaneous vagina delivery

The hazard rate of under-5 mortality was significantly higher in the younger women when compared to mothers within the age range of 45 to 49 years. After adjusting for all the observed variables in the study including country, the hazard rate for the current age group of the mothers were 15–19 years (HR: 1.59, 95% CI: [1,54-1,64]), 20–24 years (HR: 1.43, 95% CI: [1.37–1.49]), 25–29 years (HR: 1.32, 95% CI: [1.27–1.40]), 30–34 years (HR: 1.11, 95% CI: [1.09–1.15]), 35–39 years (HR: 1.09, 95% CI: [1.05–1.13]), 40–44 years (HR: 1.08, 95% CI: [1.02–1.18]) when compared to those in the age range of 45–49 years. When compared to mothers in the age range of 45–49 years, the adjusted hazard rate for those in the age range 15–19 years was significantly higher in Burkina Faso (HR: 1.8, 95% CI: [1.28–2.23]), Cote D’lvoire (HR: 1.23, 95% CI: [1.07–1.41]), Gambia (HR: 4.51, 95% CI: [3.28–6.03]), Guinea (HR: 1.78, 95% CI: [1.29–2.40]), Sierra Leone (HR: 1.95, 95% CI: [1.58–2.40]) and Togo (HR: 1.93, 95% CI: [1.29–2.74]) whilst it was significantly lower in Nigeria (HR: 0.69, 95% CI: [0.65–0.75]) and Niger (HR: 0.45, [0.37–0.54]).

In the combined data analysis, after adjusting for observed variables in the study, mothers with tertiary level of education had a lower significant adjusted hazard rate of 18% compared to those with no formal education. The risk of under-five mortality for mothers with primary level of education as well as secondary education compared to no education were both significantly lower at 9 and 27% respectively. Within country level analysis, the adjusted hazard rates were significantly higher in Sierra Leone and the Gambia, whilst it was significantly lower in Benin, Cote D’Ivoire, Liberia, Niger and Nigeria for mothers with primary, secondary and tertiary education as against mothers with no formal education.

The hazard rates were significantly lower (10%) for mothers who were currently unemployed compared to those who were currently employed. For country specific, Gambia recorded a lower percentage of 22%, whilst Guinea, Mali and Sierra Leone recorded a significantly lower hazard rate of 41, 26 and 41% respectively among those who were currently unemployed compared to those who were currently employed. However, in Benin and Liberia, the adjusted under-5 mortality rate was significantly higher, that is 21 and 11% respectively among those who were currently unemployed compared to those who were currently employed.

A significantly lower hazard rate for mothers who were currently married or in a union compared to those who were not currently married or in a union in the overall country analysis was observed to have lower hazard risk 0.79, (95% CI: 0.74–0.83). Similar rates were estimated within all the eleven of the twelve West African countries except for Cote D’lvoire for which marital status was not a significant factor of under-5 mortality rate.

Mothers residing in rural areas were 45% more likely to experience under-five mortality compared to those residing in urban areas in the overall country analysis (HR: 1.45, 95% CI: [1.37–1.55]). Similar higher significant hazard rate of under-5 mortality were estimated in Benin (HR: 1.82, 95% CI: [1.57–2.09]), Cote D’Ivoire (1.44, 95% CI: [1.21–1.68]), Mali (HR: 1.77, 95% CI: [1.41–2.14]), Nigeria (HR: 1.34, 95% CI: [1.23–1.46]) and Niger (HR: 5.00, 95% CI: [4.49–5.52]) for mothers in rural areas compared to those in urban areas. Gambia (HR: 0.16, 95% CI: [0.12–0.20]) and Guinea (HR: 0.62, 95% CI: [0.51–0.73]) were the only countries to have significantly lower hazard rates of under-5 mortality for mothers in rural areas compared to those in urban areas.

Multiple birth children were about 3 times significantly higher compared to singleton birth children, in the overall country analysis (HR: 2.81, 95% CI: [2.60–3.02]). With the exception of children from Gambia, Nigeria and Sierra Leone, the hazard rates were significantly higher in the remaining nine West African countries.

We observed significantly higher hazard rates (14%) for the male compared to the female children in the overall country analysis (HR: 1.14, 95% CI: [1.10–1.18]). The country specific analysis in Benin (HR: 1.36, 95% CI: [1.20–1.54]), Cote D’lvoire (HR: 1.18, 95% CI: 1.04–1.34]), Guinea (HR: 1.22, 95% CI: [1.07–1.39]), Liberia (HR: 1.16, 95% CI: [1.01–1.31]), Mali (HR: 1.25, 95% CI: [1.04–1.47]) and Nigeria (HR: 1.10, 95% CI: [1.05–1.16]) showed higher adjusted under-5 mortality hazard rates among male children compared to female children, whilst Niger (0.88, 95% CI: [0.81–0.94]) was the only country to report significantly lower hazard rate of males compared to females. Children who were delivered by unskilled birth attendants had significantly higher mortality (15%) compared to those who were delivered by skilled birth attendants in all the country analysis (HR: 1.15, 95% CI: 1.08–1.22]). These are shown in Table 2.

### Explaining the gamma parameter and the unobserved effects

Estimation of the gamma parameter of the Gompertz distribution for each of the countries range from − 0.04 to − 0.07. This shows that over a period, there is a decreased risk of mortality among under-fives. Similar observation was made with the combined data set among all the twelve countries. The posterior mean and its credible interval were − 0.04 (− 0.4, − 0.03) indicating that the gamma parameter is statistically significantly different from zero. The variance explaining the unobserved effect is represented by the ln (theta) parameter. This parameter was estimated taking into consideration the variation between communities or clusters or enumeration areas for specific countries and the overall. The ln (theta) is the shared parameter of the under-fives indicating that those grouped into the same cluster may have similar characteristics or share the same frailty but differ between or from cluster to cluster. In other words, the probability of under-five mortality may be similar within a cluster but different between clusters due to some characteristics that were not or could not be measured. We observed a country specific and overall statistically significant difference of the unobserved effect, implying significant difference for probability among under-fives from cluster to cluster.

## Discussion

This study investigated country specific prevalence of under-five mortality across twelve out of the eighteen West African countries for which data were available. The study further looked at the determinants of under-five mortality rates per 1000 livebirths across all these countries and also with the combined data. Data for this current study were obtained from 2012 to 2015 country specific Demographic and Health Surveys (DHS).

To effectively reduce under-five mortality in the world and more specifically in the West African sub-region, it’s important to know the factors that either contribute positively or negatively to it. This will inform policy makers and implementers as well as Government and non-Governmental organizations as to what to target. These factors are mostly not unique across the sub-region and therefore calls for different interventions than a holistic one. This is important because, as at 2015, Alkema et al., [30] estimated that 16,000 children die every day which was equivalent to 11 deaths per every minute. This implies that if causes are not determined and measures taken to drastically reduce under-five mortalities about 68.8 million children are likely to die before their fifth birthday by 2030.

A number of studies have looked at factors associated with under-five mortalities in the literature using DHS data from some of the countries included in our analysis but without considering the importance that communities (clusters) play in accurately estimating these factors. In this study, we have only not determined socio-economic and demographic factors associated with under-five mortality but gone further to look at the importance of community variations in relation to under-five mortality. Therefore, all estimates were determined for under-five mortality jointly by the individual socio-demographic and socio-economic as well as the unobserved community level effects. At the preliminary analysis, family or household effect was insignificant and was therefore dropped.

The multivariate analysis was carried out using the Gompertz model with Gamma frailty approach. The Gompertz gamma frailty model was arrived at after a comparison was made with other parametric gamma frailty models as illustrated earlier in the methods section. Discrimination and final selection of the best model (Gompertz) for this dataset was made using Bayes factor and deviance information criteria. The results showed a statistically significant community level effect on the risk of children dying before the age of five and also demonstrated variations from community to community. This approach is similar to those carried out by other researchers, for example Griffiths et al. 2004 [31], Madise et al. 1999 [32], Sahu et al. 2000 [33] and Van de Poel et al. 2009 [34].

There has being a significant progress made over the last 25 years to improve the global survival rates of children under-five. It is estimated that, worldwide there has been a 53% decline in under-five mortality from 1990 to 2015 resulting in a drop of 12.7 million deaths to about 5.9 million. Though these figures suggest a significant decline in under-five mortalities, there exist variant decline rates among all the twelve West African countries that are included in this current work. Burkina Faso (124.4), Cote D’lvoire (110.1), Guinea (116.4), Nigeria (120.6) and Niger (118.3) recorded the highest under-five mortality rates per 1000 livebirths. The lowest mortality rates were recorded in Gambia followed by Ghana and then Benin at (48.1), (60.1) and (70.4) per 1000 live births respectively. None of these West African countries met the then Millennium Development Goal 4 (MDG4) of 2015. A study by Alkema et al., [30] stipulates that, despite these reductions in under-five mortalities in sub-Saharan Africa, the projected MDG 4 target which was supposed to be met in 2015 will be met in 2026 if trends from 2015 continue.

Further analysis and observations from this current work suggest that type of birth (multiple) recorded the highest under-five mortality rate in all the 12 countries. For instance, Sierra Leone and Burkina Faso recorded 298 and 289 per 1000 livebirths respectively if the type of birth was multiple.

Our results suggest that quite a number of variables in determining under-five mortalities differ in some of the West Africa countries significantly. While in some countries certain variables increase the risk, in other countries those same variables lower the risk of under-five mortality. We observed that married mothers are less likely to experience under-five mortality compared to mothers who are not married. This could be as a result of support they receive from their husbands in taking care of their children. These findings are similar to that reported in Yaya et al. [35]. When country as variable was included in the analysis with Ghana as the reference category, it was observed that countries such as Benin, Cote D’ Ivoire, Gambia and Guinea had significantly lower risk of under-five mortalities. The rest of the remaining seven countries had higher risk ranging from 19 to 86% of experiencing under-five mortalities compared to Ghana.

Quite a number of variations across countries with respect to determinants were observed. While in Gambia, Guinea, Mali and Sierra Leone unemployed mothers had a lower risk of experiencing under-five mortalities, countries like Benin and Liberia had a higher risk instead. Also observed with variate risk was place of residence. Mothers who resided in rural areas had a statistically significant higher risk for all the twelve West African countries except Gambia and Guinea that the reverse was the case. Higher risk of under-five mortality for rural residence was also reported by Van de Poel et al. [34]. Though it is widely reported that residing in a rural community increases the risk of experiencing under-five mortality, this conclusion differs across some countries as stipulated above and is supported by the works of Fotso et al. 2007 [36]; Garenne [37]. This scenario may be as a result of unplanned urbanization and or an increasing rate of urban poverty or worsening economic situation for people living in the urban areas. Unplanned urbanization results in uncontrollable poor environmental problems in these countries that leads to high burden of diseases.

Mothers with multiple births were more than twice likely to suffer under-five mortality among all except Gambia and Guinea where lower risk was instead recorded. This finding is supported by a study Akinyemi et al. [38] conducted in Nigeria using the Nigeria Demographic and Health Surveys from 1990 to 2008. They observed an increased risk of under-five mortality among multiple births.

With sex of the child, only Niger recorded a higher risk of under-five mortality among females compared to males but the rest of countries recorded the opposite, which is higher risk for male children as against female.

Survival of children depends largely on the mother’s age at the child’s birth as was observed in this analysis. All the countries except Benin, Guinea, Liberia, Nigeria and Niger had a higher risk of mortality when the age of the mother at the time of the child birth was 15–19 compared to those of 34+ years. This could be attributable to factors such as social, economic and community support from their families. It could also be because these elderly mothers are likely to be married and will therefore receive support from their husbands. Also, the older mothers may be seen to be more experienced and matured enough to take care of the child than the younger ones. Similar findings were made by other researchers such as Ladusingh and Singh 2006 [39].

All these show that some countries may have similar or different socio-economic and demographic needs vis-à-vis intervention and implementation strategies. And so, a holistic approach across the West African sub-region in combating under-five mortalities will not inure to the benefit of all the countries.

### Strengths and limitations

Demographic and Health Survey is one of the most relied upon data in the sub-Saharan Africa in estimating and projecting individual and community indicators. Data from all DHS participating countries are standardized and fellow a similar multi-stage sampling approach. This allows for ease of comparison of results across all of these countries. All estimates were made based on the available data for each country’s Demographic and Health Surveys collected 5 years prior to the survey which are dependent upon the ability of the respondent to recollect past events and experiences. As a result, some of the information gathered may not be accurate and have the potential to bias the study results. Though comparisons were made across countries, this comparison may not be accurate considering the times that these data were collected at the individual countries. It’s therefore important that interpretations and conclusions within and across countries are done cautiously.

## Conclusions

This study made use of Demographic and Health Surveys data conducted prior to the end of the then Millennium Development Goals of 2015. It highlights the prevalence and determinants of under-five mortality across the twelve West African countries which was observed to differ significantly among the participating countries. It was also observed that quite a number of the determinants in some cases increase the rate of experiencing under-five mortality in some of the countries while in others those same variables decrease it. Though sub-Saharan Africa and more specifically West Africa have made a lot of progress with respect to reducing under-five mortality, there is still quite a substantial amount of work to be done in order to meet the Sustainable Development Goal 3 in 2030. There are variant differences among the twelve West African countries with respect to mortality rates as well as determinants which require different interventions and policy decisions.

## Data Availability

An application requesting for the use of the Demographic and Health Surveys data was sent to the DHS website. Data was then used after approval was obtained. The datasets generated and/or analyzed during the current study as well as study materials including questionnaires for the survey are available in the Demographic and Health Survey Repository, http://dhsprogram.com/data/available-datasets.cfm.

## Abbreviations

DHS: Demographic and Health Survey
MDG: Millennium Development Goals
CI: Credible Interval
AIC: Akaike Information Criteria
BIC: Bayesian Information Criteria
DIC: Deviance Information Criteria
BF: Bayes Factor
ML: Marginal Likelihood
HR: Hazard Rate
